# Advancing healthcare simulation research: innovations in theory, methodology, and method

**DOI:** 10.1186/s41077-022-00219-y

**Published:** 2022-07-27

**Authors:** Walter Eppich, Gabriel Reedy

**Affiliations:** 1 RCSI SIM Centre for Simulation Education and Research, RCSI University of Medicine and Health Sciences, Dublin, Ireland; 2 Faculty of Life Sciences and Medicine, King’s College London, Waterloo Bridge Wing G7, London, UK

Modern healthcare simulation has expanded rapidly over the past 20 years, and during that time the field has grown, developed, and changed significantly. From its origins in resuscitation education [1], anaesthesia [2, 3], and emergency medicine [4], healthcare simulation has expanded to other specialities, disciplines, and professions. Increasingly, simulation has been adopted as a replacement for clinical practice in pre-registration training [5], a trend only accelerated by the COVID-19 pandemic [6, 7]. We have also seen an expansion of simulation modalities, from a focus on ‘high-fidelity’ mannequin-based simulations [8] to simulated patient methodology [9] to hybrid simulations that integrate various simulation modalities [10]. More recently, innovative educators have applied simulation in mental health and social work [11] and simulation practitioners have identified latent threats to patient safety through in situ simulation [12].

How will we continue to push boundaries and generate new insights and innovations in the coming decades? Research has already begun to show the impact of simulation-based strategies both on learners and patients. Further, we have used simulation as a methodology to study other phenomenon in reproducible ways [13, 14]. Collectively, our field has begun to turn its attention away from ‘if’ simulation works towards ‘how’, ‘under what conditions’, and ‘to what end’? Indeed, we need to continue the move away from ‘justification’ and towards ‘clarification’ research [15]. At this juncture, a moment of collective reflection seems timely: will today’s prevailing research methods, and their methodological and theoretical underpinnings, help simulation lead us into the future of healthcare?

Some conceptual and theoretical frameworks have served the field well, and shaped significant bodies of research and scholarship. For example, ‘experiential learning’ [16], ‘reflective practice’ [17], ‘mastery learning’ [18], and Kirkpatrick’s framework for training outcomes [19] have illuminated important aspects of simulation-based education and have advanced our field. As in any field, however, these frameworks have created dominant discourses focused on particular areas of study and approaches to inquiry, while others remain fallow and demand further exploration. Lingard (2009) notes how language shapes “what we see and don’t see” [20], highlighting how discourses may limit our sense of possibility, and by extension, the nature of the science in which we engage and the knowledge that science generates.

As a research enterprise, our field must extend our scholarly inquiry in three distinct but mutually connected ways, each building on the others. Firstly, we must become more thoughtful about engaging with and integrating relevant theoretical perspectives in our work, and starting to more actively include those that still remain underrepresented in the field. This requires us to be more explicit in demanding clear and conceptually sound integration of theory in our scholarship. Several common approaches to applying theory in educational scholarship are, despite their prevalence, insufficient: for instance, dropping theory in the introduction without further reference, or mentioning diverse theories without clarifying their significance for the study design [21]. We must explicitly integrate theory as a conceptual and framing device within our work to inform research design and analysis [21, 22]. While arguably this practice is underdeveloped across much research in health professions education [23], several solidly grounded programmes of simulation-based work help guide us in this direction: for example, work on mastery learning [24, 25], directed self-regulated learning [26–28], and rapport management in debriefing [29]. Further, as Bordage aptly noted: “moving the field forward is a commute between theory building and theory testing, not simply one or the other” (S127) [30]. Thus, we must engage dialogically with theory, not only using it to frame our work, but also to generate, build, and contribute to theory through programmes of research.

Secondly, and building on the first, we must identify innovative methodologies that span the breadth of research paradigms and embrace them as part of mainstream healthcare simulation research. Innovative methodological approaches, much like new theoretical or conceptual frameworks, challenge our ways of thinking and knowing in healthcare simulation and allow us to widen the aperture of scholarship and research in the field. Recent work using narrative analysis [31], institutional ethnography [32], functional resonance analysis [33], and temporal task analysis [34] shown the potential for different methodological lenses to help us understand simulation—and healthcare practice—more deeply. Indeed, different methodologies also help to more accurately reflect and support emerging and innovative simulation practice: as the contexts, settings, and situations in which simulation are used continue to expand, we must consider how new and appropriate methodologies help us to understand them.

Finally, as research methods builds upon theoretical and methodological foundations, we must broaden our repertoire of both data collection methods and outcome measures. From its origins as an educational approach, early research efforts sought to justify simulation as a modality. Much foundational healthcare simulation research focused on measures of learning for clinical practice and for the development of surgical skills. Thus, many of these early studies used interventional designs such as randomized controlled trials and crossover designs [35]. As the field evolves and the focus of simulation research shifts from justification to clarification [15], qualitative methods of data collection have advanced our understanding of how simulation works and under what conditions. The widening availability of technologies for easier collection and generation of varied forms of data has opened up new possibilities: for example, eye-tracking [36] and wearable technologies help to measure movement and physiological responses in much less intrusive ways than was possible even a decade ago [37].

Rigorous research and scholarship across domains uses theory to conceptualize, frame, and ground research and to help inform research questions, methodological approaches, and data collection and analysis. Research findings then contribute back to theory, by explaining more about the phenomena under study, however that is framed. Alignments between theory, methodology and methods lead to findings with theoretical and methodological rigour; such rigour, when integrated into thematic, sustained, and cumulative research programs, characterizes disciplines such as psychology, anthropology, and sociology, as well as fields of study such as implementation science.

We can aspire similarly in healthcare simulation, but only with collective ambitions. Similar advances in healthcare simulation research would help our field further mature by clarifying vital aspects of simulation practice, including a more nuanced understanding of how it works and under what conditions. These clarifications will likely alter how we implement simulation-based education and accelerate expertise development – and ultimately, improve patient care. Such developments seem particularly important as we shift from time-based to competency-based health professions education. The potential contributions of simulation research to theory will in turn inspire and inform our field, helping us more deeply explain and understand the worlds in which we live, work, and practice.

## Data Availability

Not applicable.
